# Respiratory Symptoms and Pulmonary Functions of Workers Employed in Turkish Textile Dyeing Factories 

**DOI:** 10.3390/ijerph9041068

**Published:** 2012-03-26

**Authors:** Sibel Ozkurt, Beyza Akdag Kargi, Murat Kavas, Fatma Evyapan, Göksel Kiter, Sevin Baser

**Affiliations:** Medical Faculty, Pamukkale University, Denizli 20100, Kinikli, Turkey; Email: bakdag@pau.edu.tr (B.A.K.); kavas_murat@yahoo.com (M.K.); ffisekci@yahoo.com (F.E.); gokselkiter@yahoo.com (G.K.); basersevin@yahoo.com (S.B.)

**Keywords:** textile dyes, respiratory symptoms, pulmonary function test

## Abstract

Dyes are known to be a causative agent of occupational asthma in workers exposed to them. We have evaluated respiratory symptoms among textile workers. The study population comprised 106 exposed workers and a control (unexposed) group. Data were collected by a questionnaire. PFTs (Pulmonary Function Test) were performed. Among the exposed workers 36.8% defined phlegm. Respiratory symptoms were not significantly different between two groups. The employment duration of the exposed workers with phlegm was longer than those without phlegm (*p* = 0.027). The mean % predicted of FEF_25–75_ of the exposed workers was found to be significantly lower than the control (unexposed) group (*p* = 0.01). Our study suggests that textile dyeing might cause respiratory symptoms in workers.

## 1. Introduction

Dyes, especially reactive ones, are known to be a causative agent of occupational asthma, rhinitis and dermatitis in workers exposed to them [1,2,3,4]. Docker *et al.* showed that more than 15% of workers handling reactive dyes had work-related respiratory or nasal symptoms and considered that the symptoms could be attributed to an irritant response to chemicals used in this industry, including hydrochloric acid vapor, sulfur dioxide, as well as the reactive dyes themselves [5].

There are numerous publications on the effect of dust on the respiratory system of textile workers employed in processing textile materials such as cotton, hemp, flax and wool [6,7,8,9,10,11,12,13,14]. However, there are few available studies on respiratory function in the workers employed in the textile dyeing industry [15]. Viegi *et al.* evaluated respiratory functions in workers of a dyeing factory and found the prevalence of chronic bronchitis and dyspnea to be 32%, flow rates were significantly lower than reference values [16].

We planned this study to evaluate chronic respiratory symptoms among textile workers exposed to textile dyes and compare the respiratory symptoms and Pulmonary Function Test (PFT) results of exposed workers with a control group who were not exposed to textile dyes at work. To the best of our knowledge, this is the first study in our country (Turkey) which evaluates respiratory symptoms and PFTsof textile workers exposed to textile dyes.

## 2. Material and Methods

### 2.1. Background

This study was conducted at textile dyeing factories. Among the textile dyeing factories located in the Denizli Industrial Zone, which has over a hundred textile factories, three of them which have given permission for the study were included in the research. These factories were experts in textile dyeing, and had over two hundred workers at each. 

### 2.2. Patients and Study Design

Data were collected from 106 exposed workers and 106 in the control (unexposed) group (only working in the managerial departments of the textile dyeing factories). To avoid collection bias, all data for the exposed workers, including questionnaires, were collected by two experienced pulmonologists. Data on demographics, episodes of wheezing or chest tightness, symptoms of dyspnea, cough, phlegm, any other allergic and/or respiratory symptoms, duration of symptoms, past medical history (Are there any pulmonary diseases which were diagnosed by a doctor in the past? and pulmonary diseases that were not particularly investigated but might be were related to their occupational history, were identified by a questionnaire), smoking habits were determined by a questionnaire modified from the American Thoracic Society Questionnaire [17]. The questionnaire was administered in a person to person interview.

Pulmonary function tests were performed with a portable spirometer (MIR Spirobank) according to American Thoracic Society criteria while the patients were at rest and seated in the upright position. A minimum of three satisfactory forced expiratory manoeuvers was required of each subject. A satisfactory test required that the forced vital capacity (FVC) and forced expiratory volume in 1 second (FEV_1_) of two manoeuvers was reproducible within 5% [18]. Analyses were performed on the largest FVC and FEV_1_ expressed as percentage of the predicted value. The FVC, FEV_1_ and forced expiratory flow at 25% to 75% of the FVC (FEF_25–75_) and peak expiratory flow (PEF) were determined.

Non-smokers were defined as those who had never smoked regularly, smokers were currently smoking at least one cigarette daily, ex-smokers were those who had formerly smoked regularly but gave it up at least 6 months before the study. Control (unexposed) subjects (n = 106) were employees in the managerial departments of the textile dyeing factories and had similar age, sex, smoking habits, social and economic status. The same questionnaire and PFTs were administered to the control group.

### 2.3. Textile Dyeing Procedure

The dyeing was performed in several large open vats located in a large area with a temperature of 60 °C to 80 °C. The cotton materials were first sorted by type and then manually placed into vats and boiled for one hour.

The dyeing process used different types of commercially available azo and reactive dyes in addition to many other chemical agents. The dyes included: reactive dyes; azo and anthraquinone derivatives. The dyes were purchased from Germany.

Before dyeing, the materials are treated with acetic acid (CH_3_COOH), formic acid (HCOOH), sodium hydroxide (NaOH), sodium hydrosulphide (NaHS). At high temperatures vapors of different agents are released, including hydrogen (H_2_S) and nitrogen oxides (when azo dyes are used), as well as other vapors released from dyes, which may be found in the workplace atmosphere and inhaled by exposed workers. These workers were also exposed to high temperatures and to a high relative humidity in the workplace. Measurements of dusts, fumes and gases [acetic acid (CH_3_COOH), formic acid (HCOOH), sodium hydroxide (NaOH), sodium hydrosulphide (NaHS)] were not performed in the workplaces.

### 2.4. Statistical Analysis

Descriptive statistics (including Mean ± SD, frequency and percentage) were calculated for two groups separately. The difference between the means of variables in two groups was compared using Independent Samples t test and the difference between the medians of variables in two groups was compared using Mann Whitney U test. The Chi-Square test was used to compare categorical variables. The statistical significance was set at *p* < 0.05. The statistical analyses were performed with the statistical package program SPSS version 11.5.

## 3. Results

### 3.1. Characteristics of Patients

Our study population comprised 106 exposed workers (23.6% female and 76.4% male) with a mean age of 29.51 ± 0.56 years. Employment duration of the exposed workers was 65.39 ± 55.69 months. Control group (unexposed) comprised 106 workers (16% female, 84% male) with a mean age of 30.91 ± 0.78 years. The demographics and smoking data of the two groups are presented in Table 1. A significant correlation was detected between cigarette smoking and phlegm, wheezing and dyspnea (*p* = 0.005, *p* = 0.02, *p* = 0.033 respectively).

**Table 1:** The descriptive statistics (Mean ± SD, frequency and percentage) of the exposed workers and control (unexposed) group.

	Exposed workers (n = 106)	Control (unexposed) group (n = 106)	*p*
Mean age (±SD)	29.51 ± 0.56	30.91 ± 0.78	NS
Number of females	25 (23.6%)	17 (16.0%)	NS
Number of males	81 (76.4%)	89 (84.0%)	NS
Smoker	54 (50.9%)	52 (49.1%)	NS
Non-smoker and ex-smoker	52 (49.1%)	54 (50.9%)	NS
Cigarette (per-day)	6.44 ± 0.79	6.71 ± 0.84	NS
Employment duration (months)	65.39 ± 55.69	75.53 ± 73.63	NS

NS: Not significant, *p* > 0.05.

### 3.2. Respiratory Symptoms

Respiratory symptoms among the workers and control group are presented in Table 2.

Among the exposed workers 36.8% reported having phlegm. Other respiratory symptoms were 34% atopy, 27.4% wheezing, 25.5% cough and 14.2% dyspnea. These symptoms were not significantly different between the exposed workers and the control (unexposed) group. Comparison of exposed workers employment duration means by phlegm and atopy are presented in Table 3.

**Table 2:** Respiratory symptoms among the exposed workers and the control (unexposed) group.

	Exposed workers n (%)	Control (unexposed) group n (%)	*p*
Cough	27 (25.5)	23 (21.7)	NS
Wheezing	29 (27.4)	28 (26.4)	NS
Dyspnea	15 (14.2)	16 (15.1)	NS
Phlegm	39 (36.8)	41 (38.7)	NS
Atopy	36 (34.0)	39 (36.8)	NS

NS: Not significant, *p* > 0.05.

**Table 3:** Comparison of employment duration means by phlegm and atopy.

Exposed workers	Exposed workers Employment duration (month, Mean ± SD)	*p*
Phlegm (+)	76.28 ± 58.02	*p* = 0.027
Phlegm (−)	66.93 ± 69.36	
Atopy (+)	83.04 ± 73.71	*p* = 0.019
Atopy (−)	63.57 ± 59.41	
Cough (+)	65.78 ± 50.80	NS
Cough (−)	65.26 ± 57.58	NS
Wheezing (+)	71.24 ± 73.46	NS
Wheezing (−)	63.19 ± 47.75	NS
Dyspnea (+)	75.90 ± 67.88	NS
Dyspnea (−)	63.66 ± 53.68	NS

NS: Not significant, *p* > 0.05.

When the employment duration of the exposed workers with and without phlegm is compared, there was statistically significantly difference; the employment duration of the exposed workers with phlegm was longer than those without phlegm (*p* = 0.027). The duration of employment of those with atopy was found to be longer than those without (*p* = 0.019). Diagnosis of pulmonary diseases was put by only history taking in exposed workers and control group. 

Pulmonary diseases (tuberculosis, asthma, bronchitis) among the exposed workers and control group are presented in Table 4.

**Table 4:** Tuberculosis, asthma, bronchitis among the exposed workers and control (unexposed) group.

	Exposed workers (n = 10)	Control (unexposed) group (n = 7)
Tuberculosis	1	-
Asthma	1	1
Bronchitis	8	6

PFTs were performed on 106 exposed workers and 106 (unexposed) controls. The results of PFTs of the exposed workers and the control (unexposed) group are presented in Table 5.

**Table 5:** The descriptive statistics (including Mean ± SD) and the significance levels of PFTs of the exposed workers and the control (unexposed) group.

PFT	Exposed workers Actual value	Control (unexposed) group Actual value	*p*
FEV_1_ (L/sc)	3.68 ± 0.73	3.76 ± 0.55	NS
	(96.16 ± 11.75) *	(98.43 ± 11.69) *	
FVC (L)	4.31 ± 0.89	4.30 ± 0.64	NS
	(95.82 ± 12.61) *	(96.28 ± 12.52) *	
FEV_1_/FVC (%)	85.15 ± 5.94	85.84 ± 10.31	NS
	(104.13 ± 7.43) *	(106.09 ± 8.02) *	
PEF (L/sc)	7.29 ± 1.78	7.69 ± 1.60	NS
	(80.34 ± 13.97) *	(84.52 ± 16.30) *	
FEF_25–75_ (L/sc)	4.21 ± 1.13	4.46 ± 0.97	*p* = 0.01
	(88.99 ± 19.41) *	(96.31 ± 21.16) *	

* Predict values %. NS: Not significant, *p* > 0.05.

### 3.3. Pulmonary Function Test

The mean % predicted of FEF_25–75_ of the exposed workers was found to be significantly lower than the control (unexposed) group (*p* = 0.01). 

## 4. Discussion

In our study, symptoms were not significantly different between the exposed workers and the control (unexposed) group. Employment duration was higher in exposed workers with phlegm and atopy. The mean % predicted of FEF_25–75_ of the exposed workers was found to be significantly lower than the control (unexposed) group. Exposure to dyes may be a potential health hazard. Reactive dyes are common causative agents for respiratory symptoms among dyehouse workers [4].

Workers employed in textile dyeing industries may have developed acute and chronic respiratory symptoms. In a study, prevalences of chronic respiratory symptoms in exposed workers were significantly higher than in control workers [15]. Park *et al.* reported that 25.2% of reactive-dye exposed workers had work-related lower respiratory symptoms [19]. In a survey which was conducted at 15 textile plants with dyehouses in western Sweden, 162 workers were exposed to reactive dyes and 10 of these (6%) reported work-related respiratory symptoms [4]. In our study, there was no significant difference in respiratory symptoms (cough, wheezing, dyspnea) between the control and study group.

Jiin *et al.* showed that cough is higher in workers when compared to unexposed workers (29% and 14.8% respectively) [20]. According to Wang *et al.* symptom incidence at 3 months was 3.6%, for usual with phlegm, and 7% for usual dry cough [21]. High ECH exposure and low ECH exposure were significantly associated with cough plegm, chest tightness and dyspnea [20]. In our study, we also found cough symptoms in a similar ratio. Jiin *et al.* showed a linear relation between exposure and cough and sputum. In the study, the most common symptom was sputum and that symptom was seen more in the exposed workers [20]. 

In our study, 36.8% of the exposed workers defined phlegm. Other respiratory symptoms were 34% atopy, 27.4% wheezing, 25.5% cough and 14.2% dyspnea. These symptoms were not significantly different between the exposed workers and the control (unexposed) group in our research. This might be due to the exposed workers and the control (unexposed) group had similar smoking habits, mean age and the employment durations of both two groups were similar and the “healthy workers effect” (a phenomenon observed initially in studies of occupational diseases, whereby workers usually exhibit lower overall death rates than the general population because severely ill and disabled people are typically excluded from employment). 

Raza *et al.* have suggested that morbidity among textile workers is due to smoking [22]. Zuskin *et al.* found that the exposed nonsmoking workers had more complaints than the controls who were nonsmokers, and the respiratory symptoms were exacerbated by cigarette smoking [15]. However, Luo *et al.* showed that there was no significant relation between small airway abnormalities or obstructive lung abnormalities and smoking status [20]. In our study a significant correlation was detected between cigarette smoking and phlegm, wheezing and dyspnea. We could not analyse the effect of cigarette smoking by excluding other factors, as our study group was small and our study control group had a similar smoking history. 

After both short and long term exposures non smoking dyeing workers, primarly women, had significantly greater prevalence of dyspnea than controls [15]. Zuskin *et al.* reported that there were significantly higher prevalences of all chronic respiratory symptoms compared to the control workers among the male dyeing industry workers, and for the female dyeing workers, the differences were significant for dyspnea, rhinitis, sinusitis [15]. In another study, there were no significant differences in the prevalence of respiratory symptoms between men and women [23]. Females had significantly more complaints of chest tightness and dyspnea [20]. In our study gender results were similar for the study and control groups.

A study by Zuskin *et al.* showed that in workers exposed for more than 10 years, there were significantly higher prevalences of chronic cough and chronic phlegm in smokers than in nonsmokers [15]. Our finding was that employment duration was higher for exposed workers with phlegm and atopy. We didn’t discuss atopy, as we could not find that symptom in the literature. 

According to Pauluhn *et al.* the predominant dysfunction appeared to be consistent with obstructive rather than restrictive lung diseases [24]. Exposure and the onset of symptoms and objective evidence suggested that these symptoms were related to airflow limitation [2]. Workers demonstrated significant decreases in all ventilatory capacity tests in males and FEF_50_ and FEF_25_ for female workers [15]. Twenty-eight of 79 (35.4%) ECH exposed workers had obstructive or small airway lung lesions [20]. FEF_25–75_ may be used as a marker of initial bronchial damage [25]. In our study, the mean % predicted of FEF_25–75_ levels was significantly lower in the exposed workers. The Zuskin *et al.* Study environmental measurements were carried out at work place. Mesurements included fumes and gases, total and respirable dust samples [15,22]. 

## 5. Conclusions

To the best of our knowledge, this is the first study in our country (Turkey) which evaluates respiratory symptoms and PFTs of exposed workers who work in textile dyeing factories. Our study suggests that textile dyeing might cause some respiratory symptoms in exposed workers. It seems clear that exposure to reactive dyes should be considered a potential health hazard. 
